# Reconstructing the Intrinsic Statistical Properties of Intermittent Locomotion Through Corrections for Boundary Effects

**DOI:** 10.1007/s11538-020-00848-2

**Published:** 2021-02-17

**Authors:** Luca Cocconi, Alexander Kuhn-Régnier, Malte Neuss, Ana B. Sendova-Franks, Kim Christensen

**Affiliations:** 1 Center for Complexity Science, Imperial College London, London, SW7 2AZ UK; 2 Theoretical Physics of Biology Laboratory, The Francis Crick Institute, London, NW1 1AT UK; 3 Blackett Laboratory, Imperial College London, London, SW7 2AZ UK; 4 Technische Universität München, 80333 Munich, Germany; 5 School of Biological Sciences, University of Bristol, 24 Tyndall Avenue, Bristol, BS8 1TQ UK

**Keywords:** Intermittent locomotion, Boundary effects, Movement ecology, Intrinsic versus extrinsic movement bias, 62P10, 92D50

## Abstract

Locomotion characteristics are often recorded within bounded spaces, a constraint which introduces geometry-specific biases and potentially complicates the inference of behavioural features from empirical observations. We describe how statistical properties of an uncorrelated random walk, namely the steady-state stopping location probability density and the empirical step probability density, are affected by enclosure in a bounded space. The random walk here is considered as a null model for an organism moving intermittently in such a space, that is, the points represent stopping locations and the step is the displacement between them. Closed-form expressions are derived for motion in one dimension and simple two-dimensional geometries, in addition to an implicit expression for arbitrary (convex) geometries. For the particular choice of no-go boundary conditions, we demonstrate that the empirical step distribution is related to the intrinsic step distribution, i.e. the one we would observe in unbounded space, via a multiplicative transformation dependent solely on the boundary geometry. This conclusion allows in practice for the compensation of boundary effects and the reconstruction of the intrinsic step distribution from empirical observations.

## Introduction

Recent theoretical work on animal movement focuses attention on boundary effects at different scales, for example, population edge effects where two habitats conjoin (Potts et al. 2016), random walks in confined spaces (Bearup and Petrovskii 2015), insects following walls in circular experimental arenas (Jeanson et al. 2003), and the efficiency of catching ground-dwelling arthropods depending on trap shape (Ahmed and Petrovskii 2019). When the movement range of the studied organism is of the same order as the arena size, the boundary could affect many of the recorded locomotion characteristics. Such external bias requires theoretical models to provide expected values for intrinsic locomotion characteristics under the null hypothesis that the individual’s movement follows a simple random walk. Failure to compare the empirical values against those expected under the correct null hypothesis could lead to the wrong conclusions. For example, an organism might appear to have an intrinsic movement bias when, in fact, it follows a simple random walk.

In studying the anxiety levels in animals, such as mice, for instance, it is common practice to measure locomotion on a grid in terms of displacements and their respective frequencies (Michel and Tirelli 2002). To reduce the interference of the experimenter, these tests tend to be conducted in enclosures such as cages and monitored using cameras (Kas and Olivier 2008). Locomotion characteristics are also among the main measurements of explorative behaviour across animal species, and these are often recorded within bounded spaces (Russell et al. 2010; von Merten and Siemers 2012; Degen et al. 2015). However, as demonstrated in a recent paper by some of the present authors (Christensen et al. 2020), confining the space available to the test subject affects the statistical properties of the movement in a non-trivial, geometry-specific way, which can lead to erroneous conclusions about the existence of an inherent bias. Similar effects were observed, for example, in an experiment by Mallapur et al. (2009), who found that a decrease in the enclosure size of domestic fowl leads to a decrease in the mean and maximum step length as well as the total distance travelled. Even in the wild, many species are observed to move within ranges or home territories (Giuggioli et al. 2011; Potts and Lewis 2014; Riotte-Lambert et al. 2015). In theory, the relevance of boundary effects could be diminished by selecting data produced ‘sufficiently far’ from the boundaries. However, this is not always possible and the difficulty of estimating a suitable cut-off distance will inevitably introduce errors of unknown size into the statistics.

These effects are not limited to ecology and the study of animal behaviour but appear in a wide range of contexts. A relevant example from biophysics is the motion of molecules undergoing totally confined diffusion in cell membranes. These can be modelled as random walks following a symmetric Gaussian displacement distribution confined within a cuboid. A study by Ritchie et al. (2005) found that increasing the time span over which the molecule’s displacements were averaged resulted in an increasingly peaked and circular distribution in position probabilities. This observation demonstrates that the interplay of dynamics and geometry affects the ability to infer the shape of the enclosing geometry solely from the time averaged position probability.

In the present study, we establish a firm theoretical framework for analysing the effect of an arbitrary boundary on the empirical probability density of the stopping location and step of an uncorrelated random walk (Sect. 2). Our inspiration originates in intermittent locomotion where the stopping locations are the turning points and the steps are the displacements between them. Due to their special role in locomotion, the distribution of stopping locations is not necessarily the same as the distribution of locations. For the particular choice of no-go boundary conditions, our results show how to compensate for these boundary effects and provide a procedure for the reconstruction of the unconstrained step distribution from the empirically observed one (Sect. 3). Extensions to the more realistic case of a correlated random walk are beyond the scope of the present work; however, as shown in Sects. 2.2.1 and 2.2.2, this may not be needed to understand the aforementioned observations (Mallapur et al. 2009; Ritchie et al. 2005), whose qualitative features already arise when considering uncorrelated random walks in a bounded space. This suggests that the results for the uncorrelated random walk could be used to provide estimates of boundary effects on a correlated random walk.

### Uncorrelated Random Walk

In this study, we consider a symmetric Markovian random walk, which can be interpreted as a null model for intermittent locomotion (Kramer and McLaughlin 2001), also referred to as saltatory pattern (O’Brien et al. 1990), of an individual organism with no bodily features and no underlying decision-making capacity (Schwartz 2016). A clearly defined null model is of the utmost importance in the identification of relevant (e.g. behavioural) features from empirical data, as it provides an explicit term of comparison. Since the observables of interest are static, we henceforth ignore any temporal aspect of the motion, thus reducing the random walk to a sequence of stopping locations. A sample sequence of consecutive stopping locations can be generated via the following procedure:

1. Initiate the walker at location $\mathbf {r}_{i=0}$ chosen uniformly at random within the bounded domain $\varOmega$.
2. Pick a step $\delta \mathbf {r}$ with probability $f_i(\delta \mathbf {r})$, where $f_i(\delta \mathbf {r})$ denotes the probability of observing a step $\delta \mathbf {r}$ in unbounded space. For the purpose of numerical simulations, this probability distribution is assumed to be known. However, as we demonstrate in Sect. 2, extracting it from experimental observations is not trivial.
3. If $\mathbf {r}_{i} + \delta \mathbf {r} \in \varOmega$, the random walker moves to a new location $\mathbf {r}_{i+1} = \mathbf {r}_{i} + \delta \mathbf {r}$. Otherwise, a new step is determined consistently with the particular choice of boundary conditions. This might require re-sampling the probability density $f_i(\delta \mathbf {r})$, e.g. in the case of no-go boundary conditions.
4. Steps 2–3 are repeated *N* times. Time series of stopping location coordinates and steps are recorded.

Relevant observables can be extracted from the recorded sequence. The basic procedure $1 \rightarrow 4$ is then repeated *M* times and ensemble averages are calculated. It is understood that time averages converge to ensemble averages for large enough *N*, due to the ergodic nature of the process. Finally, it is important to notice that for a fixed functional form of $f_i(\delta \mathbf {r})$, the statistical properties of the random walk depend solely on the dimensionless ratio of the system size and some relevant length scale of the step distribution (e.g. its variance). In other words, they are independent of our choice of units. Hence, we are free to fix the system size while varying $f_i(\delta \mathbf {r})$ without loss of generality.

## Step and Stopping Location Probability Density

The most straightforward statistical analyses of intermittent locomotion are the determination of the probability density functions of the empirical stopping location and displacement . The latter is also known as the movement kernel (Avgar et al. 2016). Indeed, both functions are of immediate interest for application in ecology as they are often considered to convey information about dispersal and foraging strategies (Clobert et al. 2012; Okubo and Levin 2001; Turchin 2015; Benhamou 2014; Lepš 1981). However, the boundary which is usually present in experiments affects the empirical observations in a non-trivial manner. This makes it difficult to disentangle the intrinsic motion of the test subject from these boundary effects. Assuming that the intrinsic properties of a subject’s locomotion only depend on its internal state (Bartumeus 2009; Maye et al. 2007; Anteneodo and Chialvo 2009) and not on the subject’s location within the bounded domain, it is reasonable to disregard the information about the starting point of each step and to consider only the location averaged form of the step distributions. Sometimes this approach may be necessitated by data which only informs about the displacements or their magnitudes. Consequently, one must first find general expressions for the stopping location probability.

For simplicity the effects of enclosure in a confined space upon the random walker’s empirical displacement probability density function are first investigated in one dimension (Sect. 2.1) before extending the results to arbitrary convex domains in two dimensions (Sect. 2.2, where we also briefly discuss convex domains of arbitrary dimension). For practical reasons, most experiments involving motion in enclosed spaces take place in either circular or rectangular geometries, such as a Petri dish or a cage. Consequently, Sects. 2.2.1 and 2.2.2 address these particular two-dimensional geometries explicitly.

### One-dimensional Case

We will use the phrase “step of $\ell$”, where $\ell \in {\mathbb {R}}$, to indicate a displacement between stopping locations of magnitude $|\ell |$. We say that the step is to the right (resp. left) if $\ell > 0$ (resp. $\ell < 0$). We denote by $f_i(\ell )$ the probability density function of a step $\ell$ on the whole real line, which we assume to be independent of the starting point and symmetric, $f_i(\ell ) = f_i(-\ell )$. Thus, we think of $f_i(\ell )$ as an intrinsic, or internal, characteristic of the intermittent locomotion, as opposed to the extrinsic, or external, features introduced by the interaction with the boundary. In unbounded space, $f_i(\ell )$ is the limiting form of the empirical histogram of the observed steps and can therefore be reconstructed directly from experimental data.

**Fig. 1:**
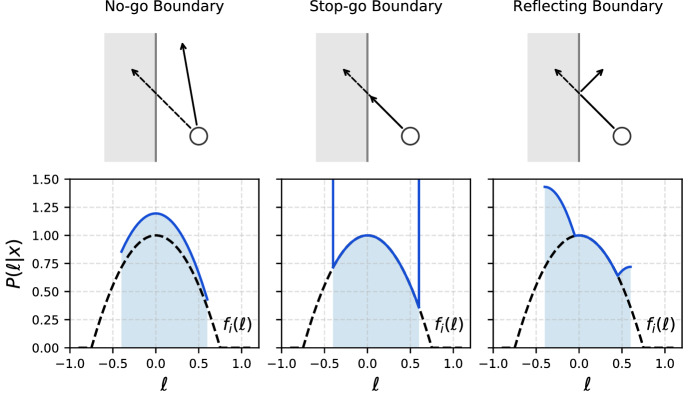
Different types of interaction with the domain boundary corresponding to different conditional probability densities $P(\ell | x)$ of the next step $\ell$ given the starting point *x*, as summarised in Eq. (1) for a random walk in one dimension (where $x \in [0,1]$). For the plots in the bottom row, we picked the intrinsic step probability density $f_i(\ell ) = 3(a^2-\ell ^2) \theta (a^2-\ell ^2)/(4 a^3)$ with $a=0.75$ and starting location $x=0.4$ (black dashed), which we compare to the conditional step probability density for each boundary condition (blue solid, shaded). Unlike the intrinsic step probability $f_i(\ell )$, the conditional probability $P(\ell |x)$ is not symmetric in general. The top part of the schematic is adapted from Bearup and Petrovskii (2015)

In bounded space, however, the probability density of a step $\ell$ differs from $f_i(\ell )$ in a boundary-condition specific way. Here, we follow the treatment of Bearup and Petrovskii (2015) and consider three types of boundary conditions: a no-go boundary, where steps extending outside of the domain are rejected; a stop-go boundary, where such steps are terminated at the boundary; and a reflecting boundary, where the portion of such steps extending beyond the domain boundary is reflected back into the domain. A schematic is provided in Fig. 1. Given a starting point at location $x \in [0,1]$, the probability density function of the next step being a step of $\ell$ terminating within the domain [0, 1] is then

1 $$\begin{aligned} P(\ell | x) = \left\{ {\begin{array}{lllll} f_i(\ell ) \theta (\ell +x) \theta (1-\ell -x) {\mathcal {N}}^{-1}(x) &{}\quad \text{ no-go } \\ f_i(\ell )\theta (\ell +x) \theta (1-\ell -x) + \delta (\ell +x) \int _{-\infty }^{-x} \mathrm{d}\ell ' f(\ell ')\\ \quad + \delta (\ell + x - 1) \int _{1-x}^\infty \mathrm{d}\ell ' f_i(\ell ') &{}\quad \text{ stop-go } \\ {[}f_i(\ell ) + f_i(\ell + 2x) + f_i(2-2x-\ell )\\ \quad + \hbox {h.o.t.}] \theta (\ell +x) \theta (1-\ell -x) &{}\quad \text{ reflecting }, \end{array}}\right. \nonumber \\ \end{aligned}$$

where $\theta$ is the Heaviside step function and $\delta$ is the Dirac delta function. The location-dependent normalisation factor ${\mathcal {N}}(x)$ appearing in the no-go case reads

2 $$\begin{aligned} {\mathcal {N}}(x) = \int _0^1 \mathrm{d}\ell ' f_i(\ell '-x). \end{aligned}$$

The higher order terms, h.o.t. in Eq. (1), entering the conditional probability for the reflecting boundary condition account for cases where multiple reflections occur in a single burst of motion between stopping events. They read

3 $$\begin{aligned} \mathrm{h.o.t.}= & {} \sum _{N=1}^\infty f_i(\ell + 2x + 2N)\nonumber \\&\quad +\,f_i(2-\ell ) + f_i(2-2x-\ell +2N) + f_i(2+\ell +2N) \end{aligned}$$

and vanish if the support of the intrinsic step probability $f_i(\ell )$ is a subset of $(-2,2)$. In a realistic setting, where the studied organism is not in distress, multiple reflections are expected to play a negligible role.

The probability density of a step of $\ell$ starting and ending in [0, 1], which we denote $f_t(\ell )$ and will henceforth refer to as the ‘transformed’ probability, is then obtained by integrating Eq. (1) over the starting location $x \in [0,1]$ with some measure $d \mu (x)$,

4 $$\begin{aligned} f_t(\ell ) = \int _0^1 \mathrm{d}\mu (x) P(\ell | x) . \end{aligned}$$

It corresponds to the limiting form of the empirical histogram of the observed steps in bounded space. In the present context, it is most meaningful to assume that the measure $\mathrm{d}\mu (x)$ corresponds to the steady-state stopping location probability density, which we denote *g*(*x*), whence $\mathrm{d}\mu (x) = g(x) \mathrm{d}x$. The probability density *g*(*x*), which depends both on $f_i(\ell )$ and on the choice of boundary conditions, can be found by solving a homogeneous Fredholm equation of the second kind of the form

5 $$\begin{aligned} g(x) = \int _0^1 \mathrm{d}x' g(x') P(x-x'|x'), \end{aligned}$$

with $P(x-x'|x')$ the conditional probability densities given in Eq. (1). For the no-go boundary condition, one can check by substitution that the integral equation (5) is solved by the ansatz $g(x) \propto {\mathcal {N}}(x)$. For this particular boundary condition, the expression for the transformed probability density function simplifies dramatically,

6 $$\begin{aligned} f_t(\ell ) \propto f_i(\ell ) (1-|\ell |) \theta (1-|\ell |) \quad \text{ for } \text{ no-go } \text{ boundary. } \end{aligned}$$

**Fig. 2:**
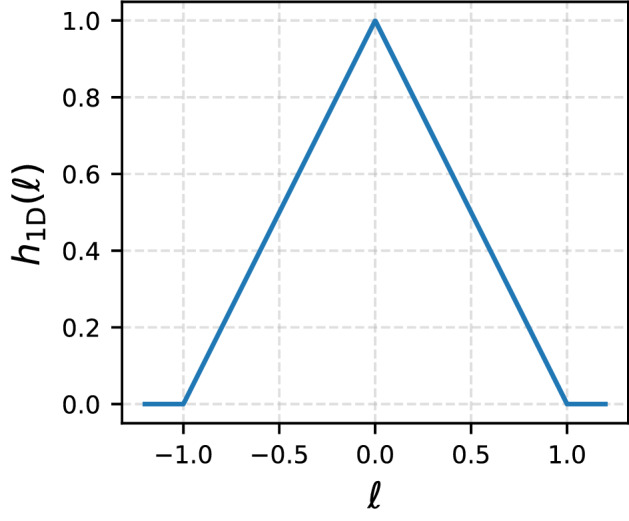
Shaper function for a simple random walk on the unit segment, $x\in [0,1]$, with no-go boundary conditions. It enters the expression for the transformed one-dimensional step probability density as a multiplicative factor in Eq. (6). This shaper function linearly suppresses the intrinsic step probability for increasing step magnitude and vanishes for step magnitudes larger than or equal to the system size

This is a remarkable result as it indicates that, for no-go boundaries, the mapping between the intrinsic step probability $f_i(\ell )$ and the empirically observed (transformed) step probability $f_t(\ell )$ is multiplicative and independent of the specific choice of $f_i(\ell )$. In particular, it only depends on the geometry of the bounded space through

7 $$\begin{aligned} h_{1D}(\ell ) := (1-|\ell |) \theta (1-|\ell |) , \end{aligned}$$

shown in Fig. 2, which we will henceforth refer to as ‘shaper’ function. We will see that this result extends straightforwardly to higher dimensions. Notably, the variance of the transformed step probability density, $\langle \ell ^2 \rangle _{t}$, is always smaller than that of the corresponding probability density in unbounded space, $\langle \ell ^2 \rangle _{i}$,

8 $$\begin{aligned} \langle \ell ^2 \rangle _{i}&= 2 \int _0^\infty \mathrm{d}\ell \ \ell ^2 f_i(\ell ) \end{aligned}$$

9 $$\begin{aligned}&\ge 2 \int _0^1 \mathrm{d}\ell \ \ell ^2 \frac{f_i(\ell )}{2\int _0^1 \mathrm{d}\ell ' f_i(\ell ')} \end{aligned}$$

10 $$\begin{aligned}&> 2 \int _0^1 \mathrm{d}\ell \ \ell ^2 \frac{(1-\ell ) f_i(\ell )}{2\int _0^1 \mathrm{d}\ell ' f_i(\ell ') (1-\ell ')} = \langle \ell ^2 \rangle _t \end{aligned}$$

where to go from Eq. (8) to (9) we have reduced the support of $f_i(\ell )$ to [0, 1] (normalising the resulting probability density accordingly) and the inequality relating Eqs. (9) and (10) follows from the shaper function being monotonically decreasing in $|\ell |$ (see “Appendix A”).

For the case of reflecting boundaries, one can check by substitution that Eq. (5) is solved by the uniform probability density $g(x)=1$, independently of $f_i(\ell )$. Substituting into Eq. (4) we find

11 $$\begin{aligned}&f_t(\ell ) = f_i(\ell ) h_{1D}(\ell ) + \int _0^1 \mathrm{d}x \ [f_i(\ell + 2x) + f_i(2-2x-\ell )\nonumber \\&\quad +\,\mathrm{h.o.t.}] \theta (\ell +x) \theta (1-\ell -x) \quad \text{ for } \text{ reflecting } \text{ boundary, } \end{aligned}$$

which lacks the simple structure that was observed for no-go boundaries.

Finally, for the case of stop-go boundaries, Eq. (5) for the steady-state stopping location probability density cannot in general be solved in closed form. To proceed further, we assume that $g(x)=1$, corresponding to an experiment where the subject is placed uniformly at random within the bounded domain and only statistics of the first step are collected. Substituting the relevant form of Eqs. (1) into (4) produces

12 $$\begin{aligned} f_t(\ell )&= f_i(\ell ) h_{1D}(\ell ) + \int _{-\infty }^\ell \mathrm{d}\ell ' f_i(\ell ') \theta (-\ell ^2-\ell ) + \int _\ell ^\infty \mathrm{d}\ell ' f_i(\ell ') \theta (\ell -\ell ^2) \end{aligned}$$

13 $$\begin{aligned}&= f_i(\ell ) h_{1D}(\ell ) + \frac{1}{2} \left( \int _{-\infty }^{-|\ell |} \mathrm{d}\ell ' f_i(\ell ') + \int _{|\ell |}^\infty \mathrm{d}\ell ' f_i(\ell ') \right) \theta (1-\ell ^2) \nonumber \\&\qquad \quad \text{ for } \text{ stop-go } \text{ boundary, } \end{aligned}$$

where in going from Eqs. (12) to (13) we have used $f_i(\ell ) = f_i(-\ell )$ to express the transformed step probability in a more symmetric fashion. Once again, the expression for the transformed step probability lacks the simple structure observed in the no-go case.

While the precise form of the transformed step probability density function depends on the combination of $f_i(\ell )$ and boundary conditions, Eqs. (6), (11), and (12) share the property that $f_t(\ell )$ vanishes for $|\ell |>1$, reflecting the impossibility for the random walker to exit the domain [0, 1]. In the following, we will focus solely on the case of no-go boundary conditions. We focus on it because we think it is compatible with the behaviour of animals that have habituated to their environment, when we do not expect many animals would have an increased probability of stopping at the boundary (the ‘stop-go’ boundary condition) or would simply be reflected off the boundary (the ‘reflecting’ boundary condition).

### Two-dimensional Case (Convex Domain)

Here, we extend the analysis presented in the previous Sect. 2.1 to intermittent locomotion in two-dimensional bounded space, focusing solely on the case of no-go boundary conditions. Displacements between successive stopping locations will be denoted ${\ell }=(\ell _x,\ell _y)$, with ${\ell } \in {\mathbb {R}}^2$. The probability density function of ${\ell }$ on the real plane is denoted $f_i(\ell _x,\ell _y)$ and is assumed to be rotationally symmetric,

14 $$\begin{aligned} f_i(\ell \cos (\theta ),\ell \sin (\theta )) = f_i(\ell ,0) \end{aligned}$$

for all $\theta \in [0,2\pi )$. Now let $\varOmega$ be a bounded convex domain and $I_\varOmega (x,y)$ be the indicator function taking the value 1 if $(x,y)\in \varOmega$ and 0 otherwise. For a convex polygon with *N* sides, such that each side $i \in \{1,\ldots ,N\}$ is given by the equation $y=a_i x + c_i$, the indicator function $I_\varOmega (x,y)$ is the following product of Heaviside step functions

15 $$\begin{aligned} I_\varOmega (x,y) = \prod _{i=1}^N \theta (q(i) J_i(x,y)) , \end{aligned}$$

where $q(i)=1$ (resp. $q(i)=-1$) if the polygon is above (resp. below) the line $y=a_i x + c_i$ and $J_i(x,y) = y-a_i x - c_i$. In bounded space and with the specific choice of no-go boundary conditions, the probability density function of the next step being a step of ${\ell }$ terminating within $\varOmega$ given a starting point at location $\mathbf {r}=(x,y)$ is

16 $$\begin{aligned} P({\ell }|\mathbf {r}) = f_i(\ell _x,\ell _y) I_\varOmega (x+\ell _x, y+\ell _y) {\mathcal {N}}^{-1}(\mathbf {r}) , \end{aligned}$$

with the normalisation factor

17 $$\begin{aligned} {\mathcal {N}}(\mathbf {r}) = \int _\varOmega \mathrm{d}\ell _x' \mathrm{d}\ell _y' \ f_i(\ell _x'-x,\ell _y'-y) . \end{aligned}$$

The reason for imposing that $\varOmega$ is convex is to avoid ambiguities when both the starting and finishing points are within $\varOmega$ but some convex combination of the two is not. The probability density of a step ${\ell }$ starting and ending in $\varOmega$, which we denote $f_t(\ell _x,\ell _y)$, is then obtained by integrating Eq. (16) over the starting location $\mathbf {r} \in \varOmega$ with some measure $\mathrm{d}\mu (\mathbf {r})$,

18 $$\begin{aligned} f_t(\ell _x,\ell _y) = \int _\varOmega \mathrm{d}\mu (\mathbf {r}) P({\ell }|\mathbf {r}). \end{aligned}$$

Similar to the one-dimensional case, we argue that this measure should correspond to the steady-state stopping location probability density function $g(\mathbf {r})$, which is defined as the solution of the integral equation

19 $$\begin{aligned} g(\mathbf {r}) = \int _\varOmega \mathrm{d}\mathbf {r}' g(\mathbf {r}') P(\mathbf {r}-\mathbf {r}'|\mathbf {r}') . \end{aligned}$$

One can check by substitution that Eq. (19) is solved by the ansatz $g(\mathbf {r}) \propto {\mathcal {N}}(\mathbf {r})$, whence

20 $$\begin{aligned} f_t(\ell _x,\ell _y) \propto f_i(\ell _x,\ell _y) \int _\varOmega \mathrm{d}x \mathrm{d}y \ I_\varOmega (x+\ell _x,y+\ell _y) . \end{aligned}$$

The mapping between the intrinsic and transformed step probability density for the case of no-go boundary conditions thus has the same structure as seen in the one-dimensional case, namely that of a multiplication by a geometric-specific shaper function of the form

21 $$\begin{aligned} h_{2D}(\ell _x,\ell _y) := \int _\varOmega \mathrm{d}x \mathrm{d}y \ I_\varOmega (x+\ell _x,y+\ell _y) . \end{aligned}$$

Assuming that $\varOmega$ is convex, the shaper function Eq. (21) is monotonically decreasing in $|{\ell }|$, the magnitude of the step (see “Appendix B”). Consequently, the variance of $|{\ell }|$ under the transformed probability density $f_t(\ell _x,\ell _y)$ is always smaller than that under the corresponding probability density $f_i(\ell _x,\ell _y)$ in unbounded space. This can be shown along the lines of Eqs. (8)–(10).

The construction outlined in this section can be extended straightforwardly to (convex) domains of arbitrary dimensionality. With $\varOmega$ a bounded *d*-dimensional convex domain and $I_\varOmega (\mathbf {r})$ the associated indicator function of $\mathbf {r} \in {\mathbb {R}}^d$, one arrives at the now familiar multiplicative relation

22 $$\begin{aligned} f_t({\ell }) \propto f_i({\ell }) \int _\varOmega \mathrm{d}\mathbf {r} \ I_\varOmega (\mathbf {r} + {\ell }) = f_i({\ell }) h_{dD}({\ell }), \end{aligned}$$

where the higher-dimensional shaper function $h_{dD}({\ell })$ is defined as

23 $$\begin{aligned} h_{dD}({\ell }) := \int _\varOmega \mathrm{d}\mathbf {r} \ I_\varOmega (\mathbf {r} + {\ell }). \end{aligned}$$

#### Square Geometry

As an example, we can use Eq. (15) for the indicator function of a polygonal domain in combination with Eq. (21) for a general shaper function to obtain the shaper function associated with a square domain of unit side, in which case

24 $$\begin{aligned} h_\square (\ell _x,\ell _y)&= \int \mathrm{d}x \mathrm{d}y \ \theta (\ell _x + x) \theta (1-\ell _x-x) \theta (\ell _y + y) \theta (1-\ell _y-y) I_\square (x,y) \nonumber \\&= (1-|\ell _x|)(1-|\ell _y|) \theta (1-|\ell _x|) \theta (1-|\ell _y|) , \end{aligned}$$

which can also be written as a product of one-dimensional shaper functions, Eq. (7),

25 $$\begin{aligned} h_\square (\ell _x,\ell _y) = h_{1D}(\ell _x) h_{1D}(\ell _y) . \end{aligned}$$

**Fig. 3:**
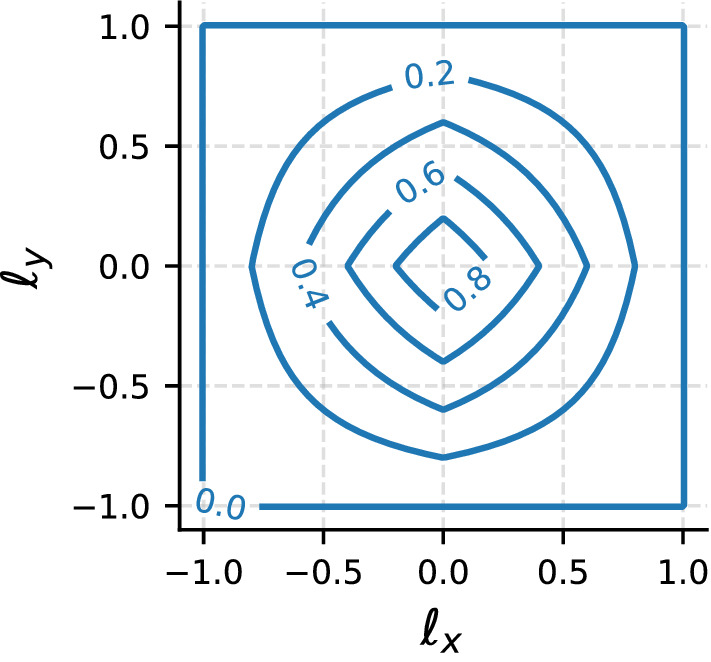
Shaper function for a square geometry of unit side as defined in Eq. (24). It vanishes in every direction when $|\ell |>\sqrt{2}$, i.e. when the step magnitude exceeds the diameter of the domain. Remarkably, the shaper function does not satisfy the same rotational symmetry of the intrinsic step probability density function, which is therefore broken in the empirical step distribution

Notably, the shaper function $h_\square (\ell _x,\ell _y)$, shown in Fig. 3, does not satisfy the same rotational symmetry as the step probability density function in unbounded space, Eq. (14), meaning that this symmetry is broken by the interaction with the boundary.

#### Circular Geometry

While the discussion above encompasses the disk of unit radius as a specific case of bounded convex domain with indicator function

26 $$\begin{aligned} I_\varOmega (x,y) = \theta \left( 1-\sqrt{x^2+y^2}\right) , \end{aligned}$$

the commonness of (at least approximately) circular boundaries in natural as well as experimental conditions makes it worthwhile presenting the corresponding results in an explicit form. Starting with the steady-state stopping location distribution, we use $g(\mathbf {r}) \propto {\mathcal {N}}(\mathbf {r})$ together with Eqs. (17), (14) and the indicator function Eq. (26) to obtain

27 $$\begin{aligned} g(\mathbf {r}) \propto \int _{0}^{1-|\mathbf {r}|} \mathrm{d}\ell \ 2\pi \ell \ f_i^{2D}(\ell ,0) + \int _{1-|\mathbf {r}|}^{1+|\mathbf {r}|} \mathrm{d}\ell \ 2\pi \ell \ f_i^{2D}(\ell ,0) \gamma (|\mathbf {r}|, \ell ) \end{aligned}$$

where

28 $$\begin{aligned} \gamma (R,\ell ) = \frac{1}{\pi } \cos ^{-1} \left( \frac{R^2+\ell ^2-1}{2R\ell } \right) . \end{aligned}$$

The geometry-specific shaper function, which we denote $h_\circ (\ell _x,\ell _y)$, can be obtained by substituting Eq. (26) into Eq. (21) and reads

29 $$\begin{aligned} h_\circ (\ell _x,\ell _y) = 2 \cos ^{-1}\left( \frac{\sqrt{\ell _x^2+\ell _y^2}}{2}\right) - \frac{1}{2}\root \of {(4-\ell _x^2-\ell _y^2)(\ell _x^2+\ell _y^2)} . \end{aligned}$$

**Fig. 4:**
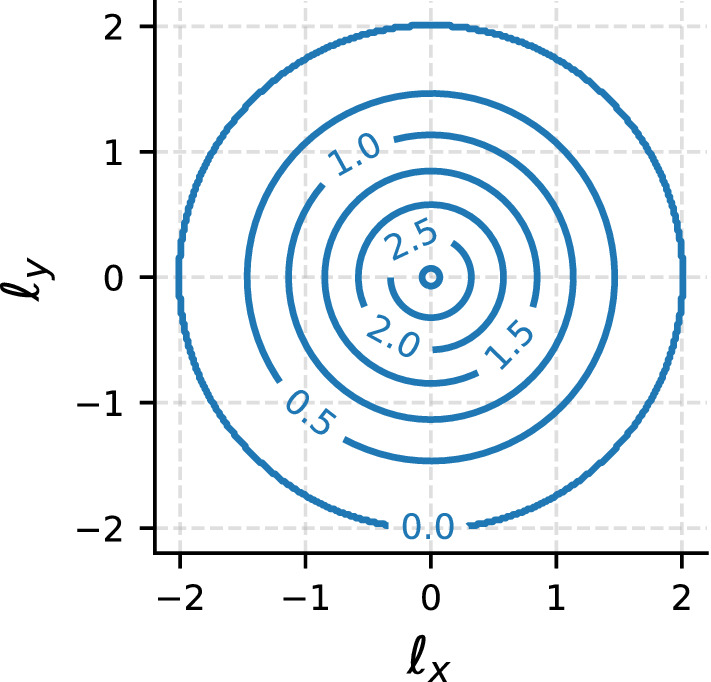
Shaper function for a circular geometry of unit radius as defined in Eq. (29). It vanishes in every direction when $|\ell |>2$, i.e. when the step magnitude exceeds the diameter of the domain. Unlike the square case, this shaper function preserves the symmetry of the intrinsic step probability density

Unlike the shaper function for a unit square domain, Eq. (24), the shaper function for a circular domain $h_\circ (\ell _x,\ell _y)$ is rotationally symmetric, see Fig. 4, and the symmetry of the intrinsic step probability density $f_i(\ell _x,\ell _y)$ is preserved under the interaction with the boundary.

#### Statistics of the Step Magnitude

In the discussion above, the dependence of the transformed step probability density function $f_t(\ell _x,\ell _y)$ on its two arguments $\ell _x$ and $\ell _y$ was left explicit. This might have been redundant for the intrinsic probability $f_i(\ell _x,\ell _y)$, which was assumed to be rotationally symmetric; however, it is not so for $f_t(\ell _x,\ell _y)$ since the shaper function $h_{2D}(\ell _x,\ell _y)$ need not satisfy that same symmetry (see Sect. 2.2.1 for an example). On the other hand, the magnitude of the displacement between successive stopping locations is often more easily accessible from an experimental point of view, as it does not require the set of coordinates to be consistent across sample paths. It is therefore instructive to consider how the probability density function of the step magnitude is affected by enclosure in a bounded space with no-go boundary conditions. We denote by ${\tilde{f}}_i(\ell )$ the probability to observe a step ${\ell }$ of magnitude $|{\ell }|=\ell$ in the real plane. It is related to its two-dimensional counterpart via

30 $$\begin{aligned} {\tilde{f}}_i(\ell ) = \int _0^{2\pi } \mathrm{d}\theta \ \ell f_i(\ell \cos (\theta ),\ell \sin (\theta )) = 2\pi \ell f_i(\ell ,0) . \end{aligned}$$

Similarly, we denote by ${\tilde{f}}_t(\ell )$ the probability to observe a step ${\ell }$ of magnitude $|{\ell }|=\ell$ starting and ending in $\varOmega$. It can be written using Eqs. (20) and (21) as

31 $$\begin{aligned} {\tilde{f}}_t(\ell )&\propto \int _0^{2\pi } \mathrm{d}\theta \ \ell f_i(\ell \cos (\theta ),\ell \sin (\theta )) h_{2D}(\ell \cos (\theta ),\ell \sin (\theta )) \nonumber \\&\propto f_i(\ell ,0) \ell \int _0^{2\pi } \mathrm{d}\theta \ h_{2D}(\ell \cos (\theta ),\ell \sin (\theta )) , \end{aligned}$$

which has the same multiplicative structure as Eq. (4) with a modified shaper function

32 $$\begin{aligned} {\tilde{h}}_{2D}(\ell ) := \int _0^{2\pi } \mathrm{d}\theta \ h_{2D}(\ell \cos (\theta ),\ell \sin (\theta )) \end{aligned}$$

depending only on the step magnitude $\ell$.

## Correcting for Boundary Effects Under No-Go Boundary Conditions

**Fig. 5:**
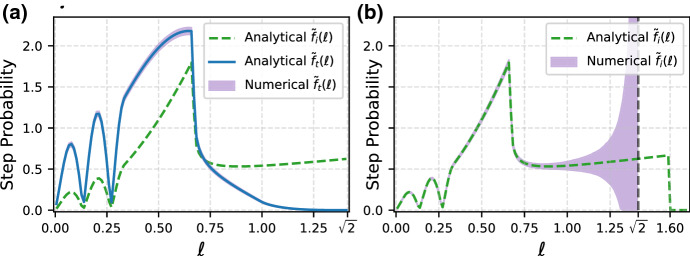
Comparison of numerical results and analytical predictions for the transformed step probability (**a**), and the reconstructed intrinsic step probability (**b**), for a square geometry with unit side. Numerical data are plotted as an envelope of one standard deviation around the mean calculated from $M=10^4$ runs of $N=10^5$ steps, the first $10^3$ of which were ignored for each run. The empirical step probability ${\tilde{f}}_t(\ell )$, purple in (**a**), is recapitulated upon multiplication of ${\tilde{f}}_i(\ell )$ by the shaper function, in agreement with Eq. (31). The uncertainty on the reconstructed intrinsic step distribution in (**b**) increases with step magnitude because larger steps are increasingly unlikely and the reciprocal of the shaper function diverges as $\ell$ approaches $\sqrt{2}$, i.e. the diameter of the domain (vertical dashed). The reconstructed intrinsic step distribution ${\tilde{f}}_i(\ell )$ in (**b**) is not normalised for ease of comparison (color figure online)

In Sect. 2, we explored how three common choices of boundary conditions (no-go, stop-go and reflecting) affect the statistical properties of intermittent locomotion in bounded space. For the particular case of no-go boundary conditions, starting from the conditional probability density function of the next step, Eq. (1) for one-dimensional domains or Eq. (16) for two-dimensional ones, we showed that the empirical step probability $f_t({\ell })$ density is related to the intrinsic step probability $f_i({\ell })$ by a multiplicative transformation involving a ‘shaper function’, Eqs. (7) or (21), that depends solely on the geometry of the domain. This simple structure can be exploited to correct for boundary effects, assuming that the boundary geometry is known. In particular, we can write

33 $$\begin{aligned} f_i(\ell ) \propto \frac{f_t(\ell )}{h_{1D}(\ell )} \quad \text{ and } \quad f_i(\ell _x,\ell _y) \propto \frac{f_t(\ell _x,\ell _y)}{h_{2D}(\ell _x,\ell _y)} \end{aligned}$$

for one- and two-dimensional geometries, respectively. When the step exceeds the maximum linear dimension of the domain in a given direction, the shaper function vanishes and the expressions in Eq. (33) become undefined. This behaviour is expected, since such steps cannot be performed inside the domain. In two dimensions and focusing on the statistics of the step magnitude, Eqs. (30) and (31) give ${\tilde{f}}_i(\ell ) \propto {\tilde{f}}_t(\ell )/{\tilde{h}}_{2D}(\ell )$, which is undefined for step magnitudes exceeding the maximum linear dimension of the bounded space. We demonstrate this procedure numerically in Fig. 5 for the case of a non-trivial intrinsic step probability density in a square geometry.

## Conclusion and Outlook

We studied the effect of enclosure in a bounded space on locomotion characteristics in a null model of an organism moving intermittently in one- and two-dimensional domains of arbitrary convex geometry. Intermittent locomotion, which characterises organisms across taxa (Kramer and McLaughlin 2001), is modelled here as a simple random walk, where we consider individual steps to represent the net displacement between stopping locations. The temporal aspect of locomotion is not taken into account. For the particular case of no-go boundary conditions, our analysis yielded analytical closed form expressions for the probability density function of the stopping location and step. Corresponding expressions were also obtained in one dimension for stop-go and reflecting boundary conditions. Both these locomotion characteristics are affected by the boundary geometry in a non-trivial way, thus demonstrating how a superficial statistical analysis can lead to erroneous conclusions. For example, the empirical variance of the step probability density function is reduced upon enclosure, which could be misinterpreted as a behavioural response to the latter.

For the particular case of no-go boundary conditions, we also demonstrated that the relation between the intrinsic step probability density, $f_i({\ell })$, and its empirical counterpart in bounded space, $f_t({\ell })$, amounts to a multiplication by a geometry-specific function of the step ${\ell }$, which we termed ‘shaper’ function and denoted $h({\ell })$. This shaper function displays a maximum at $|{\ell }|=0$ and is a monotonically decreasing function of the step magnitude $|{\ell }|$ in a given direction. In one dimension and for general two-dimensional convex domains, the shaper function can be calculated straightforwardly and often in closed form. The simple relations between $f_i({\ell })$ and $f_t({\ell })$ further enables the reconstruction of $f_i({\ell })$ from an empirically measured $f_t({\ell })$ up to the maximum step allowed within the enclosure. Any information about steps larger than this cut-off is lost, as such an observation is forbidden within the boundary. The reliability of such reconstruction has been numerically demonstrated.

The possibility of consistently eliminating boundary effects is of immediate interest in behaviour and ecology, where properties of movement can provide useful information about the state of an organism. Locomotion as performed by real organisms is generally functional to other activities (e.g. foraging) and is expected to be subjected to considerable biases. These biases can be intrinsic, such as asymmetries in body or behaviour (Wiper 2017) and changes due to learning, or extrinsic, such as the particular conformation of the bounded environment. As such, the random walk examined in this paper can be considered a null model for the displacement of an actual organism in space. Nonetheless, our study sheds light on the risks associated with overlooking boundary effects when interpreting locomotion data as a behavioural feature. Higher moments of the step probability density, particularly the correlation function of sequential steps, were not considered in this work. However, a recent study by some of the authors (Christensen et al. 2020) demonstrates how enclosure also introduces extrinsic negative correlations, which are required to prevent the random walker from exiting the domain.

## Figures and Tables

**Fig. 6:**
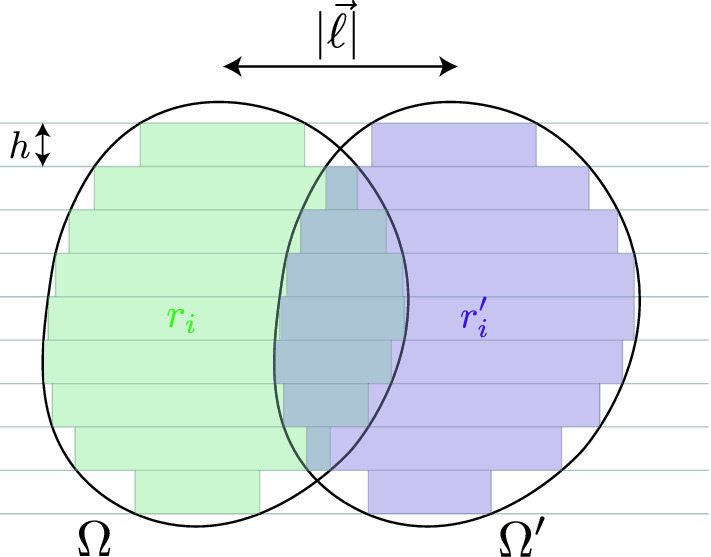
Area of the intersection of two convex domains of identical geometry, $\varOmega$ and $\varOmega '$, with a relative displacement of ${\ell }$. Each domain is approximated as a union of non-overlapping rectangular domains of height *h* (shaded), such that each rectangular sub-domain of $\varOmega$ can only ever overlap with one rectangular sub-domain of $\varOmega$ as $|{\ell }|$ varies

## Data Availability

Computer code is available at http://doi.org/10.5281/zenodo.4119100 (Kuhn-Regnier et al. 2020).

## A Variance of $f_t(\ell )$ in One Dimension

Let *F*(*x*) be a non-negative, monotonically decreasing function of *x*. Let *P*(*x*) be a probability density function of $x \in [0,1]$ satisfying the normalisation condition $\int _0^1 \mathrm{d}x \ P(x) = 1$. Then $P(x) F(x) /(\int _0^1 \mathrm{d}y P(y) F(y))$ is also a valid probability density function of $x \in [0,1]$. Define the variance of *x* with respect to the two probability densities

34 $$\begin{aligned}&I = \int _0^1 \mathrm{d}x \ P(x) x^2 = \langle x^2 \rangle \end{aligned}$$

35 $$\begin{aligned}&J = \int _0^1 \mathrm{d}x \frac{P(x)F(x)}{\int _0^1 \mathrm{d}y P(y) F(y)} x^2 = \frac{\langle F(x) x^2 \rangle }{\langle F(x) \rangle } , \end{aligned}$$

corresponding to expressions (8) and (10), Sect. 2.1. In order to prove the inequality $I > J$, we first use the law of total expectation to write

36 $$\begin{aligned} \langle x^2 F(x) \rangle = \langle x^2 \rangle \langle F(x)\rangle + \mathrm{Cov}(x^2,F(x)) , \end{aligned}$$

whence

37 $$\begin{aligned} I > J \iff \mathrm{Cov}(x^2,F(x)) < 0 . \end{aligned}$$

The proof of the right-hand side of equivalence (37) for *F*(*x*) a monotonically decreasing function of *x* is a standard textbook exercise. It follows by noticing that $\langle (F(x) - F(y)) (x^2 - y^2) \rangle < 0$ for any pair of random variables *x* and *y* and that $\langle (F(x) - F(y)) (x^2 - y^2) \rangle = 2 \text {Cov}(x^2,F)$ when *x* and *y* are i.i.d.

## B Overlap of Indicator Functions

The generic two-dimensional shaper function for convex domains $h_{2D}(\ell _x,\ell _y)$ of Eq. (21) can be interpreted as the area of the intersection between $\varOmega$ and a copy of itself, $\varOmega '$, which was offset by ${\ell }=(\ell _x,\ell _y)$. Using the indicator function,

38 $$\begin{aligned} h_{2D}(\ell _x,\ell _y) = \int _{-\infty }^\infty \mathrm{d}x \mathrm{d}y \ I_\varOmega (x,y) I_\varOmega (x+\ell _x,y+\ell _y) . \end{aligned}$$

To demonstrate that $h_{2D}(\ell \cos (\theta ),\ell \sin (\theta ))$ decreases monotonically with $|\ell |$ at fixed $\theta$, we first perform a rotation of the coordinate axes so that, without loss of generality, $\theta = 0$ and the relative offset of $\varOmega$ and $\varOmega '$ is aligned with the *x*-axis. We now approximate $\varOmega$ and $\varOmega '$ as unions of *N* non-overlapping rectangular domains $r_i$ and $r_i'$ of height *h*; see Fig. 6, with the understanding that this approximation becomes exact in the limit $h \rightarrow 0$. The intersection $\varOmega \cap \varOmega '$ can now be written as a union of intersections

39 $$\begin{aligned} \varOmega \cap \varOmega ' = \bigcup \limits _{i=1}^{N} (r_i \cap r_i') , \end{aligned}$$

whence

40 $$\begin{aligned} h_{2D}(\ell _x,\ell _y) = \sum _i \int _{-\infty }^\infty \mathrm{d}x \mathrm{d}y \ I_{r_i}(x,y) I_{r_i'}(x+\ell ,y) . \end{aligned}$$

It is straightforward to show that each term appearing in the sum in the right-hand side of Eq. (40) is monotonically decreasing in $|\ell |$. Consequently, $h_{2D}(\ell \cos (\theta ),\ell \sin (\theta ))$ also decreases monotonically with $|\ell |$ at fixed $\theta$.
